# Development and Evaluation of a Proficiency-based and Simulation-based Surgical Skills Training for Technical Medicine Students

**DOI:** 10.15694/mep.2020.000284.1

**Published:** 2020-12-17

**Authors:** Frank Halfwerk, Erik Groot Jebbink, Marleen Groenier

**Affiliations:** 1 Department of Cardio-Thoracic Surgery; 2 Technical Medical Centre; 3 Technical Medical Centre

**Keywords:** Patient-centred, proficiency-based training, simulation-based training, patient journey, preclinical learning

## Abstract

This article was migrated. The article was marked as recommended.

Objective

Surgical graduate training to achieve practice-ready students is needed, yet is often lacking. This study developed and evaluated a proficiency-based, simulation-based course for basic surgical skills at graduate level. Learning outcomes were measured at the level of knowledge and skills and evaluated with a post-course questionnaire after students’ clinical rotations.

Methods

The surgical skills course was anchored to surgical patient flow and covered topics and skills related to pre-, intra-, and post-operative care, including case-based medical reasoning, patient safety, infection management, operating theatre etiquette, scrubbing and donning, instrument handling, local anaesthesia, excision of tissue, and suturing. Students were assessed on knowledge and procedural skills.

Results

155 graduate Technical Medicine students from academic years 2015-2016 and 2016-2017 entered this 10-week, 3 ECTS credits graduate Surgical Skills course.

Pass rates of the knowledge test were 78%, and 87% for the procedural skill assessment.

Graduate students reached proficiency level in a simulation-based basic surgical skills course. Students stated to go with confidence to the operating room and felt competent in performing four basic surgical skills.

Conclusion

Based on this study, we recommend that proficiency-based training using simulation should be standard in surgical curricula before students are allowed to practice on patients.

## Introduction

### Surgical training

International reports on surgical safety reveal major deficiencies in worldwide surgical skills training ( Kohn, Corrigan and Donaldson, 2000; Eden *et al.*, 2014), which is reflected by the United Kingdom’s higher mortality rates (6%) on the first day of junior doctors ( Jen *et al.*, 2009). Surgical educators express increased concerns about a lack of confidence and competence among residents ( Bhatti, 2017). Furthermore, non-technical skills for surgeons (NOTSS) such as situational awareness, decision-making, communication and teamwork and leadership are often not assessed but evaluated implicitly ( Gostlow *et al.*, 2017). Surgical educators named performance assessment and curriculum development as most important topics in surgical education followed by simulation ( Stefanidis *et al.*, 2015a). Undergraduate medical education in a preparatory curriculum to achieve well-trained practice-ready graduates is needed, yet is often lacking ( American Board of Surgery *et al.*, 2014).

### Simulation-based training

Surgical training moves towards outcome-based performance assessment and simulation is increasingly used for that goal ( Satava, 2006; Stefanidis *et al.*, 2015b). Simulation-based training (SBT) allows for standardized teaching and objective evaluation of surgical skills ( Lammers *et al.*, 2008; Ahmed *et al.*, 2011). Ideally, SBT is proficiency-based and trainees have to master a particular skill before they progress to a more complex skill or environment ( Gallagher *et al.*, 2005). Embedding SBT in a graduate curriculum has the advantage that students attain proficiency prior to their clinical rotations or residency. Training of (surgical) skills before clinical rotations shape behaviour and compliance to guidelines as they form long-term habits during their rotations ( Erasmus *et al.*, 2020). Role models in SBT who encourage good hand hygiene compliance and good habit, might strongly influence students’ clinical behaviour. Furthermore, simulation might decrease cognitive overload for students stressfully trying to maintain sterility in the operating theatre (OR) ( Cooper and Tisdell, 2020) and benefits students by providing an environment that encourages making mistakes, learning to correct them, and reflecting on what is learned ( Satava, 2006). A lack of qualified and available staff in combination with initial high costs of developing SBT often limit incorporation of SBT.

### Assessment of surgical skills

Effects of surgical skills training can be assessed with a pre- post survey of participants ( Brunt *et al.*, 2008; Pender *et al.*, 2017), in metrics such as time to complete a task ( Brunt *et al.*, 2008; Pender *et al.*, 2017), or expert assessment ( Gawad *et al.*, 2019). Variability in evaluations could result in biased scores or unsafe patient care and undermine validity and reliability of the students’ assessment scores.

At the Technical Medical Centre of the University of Twente a compulsory surgical skill course for graduate students in Technical Medicine is given. However, internal student evaluations revealed a limited relationship between course content and current clinical practice. Also, pass/fail decisions relied on a single observation made by a single assessor which introduces bias to the decisions made. Last, educational themes in the course lacked coherence and patient’s perspective. These observations prompted us to revise the basic surgical skills course and take abovementioned strategies for successful SBT into account.

This study aims to develop a preclinical curriculum to teach 21 ^st^ century surgical skills, assess surgical skills in an objective way and evaluate this curriculum with a post-course questionnaire after students’ clinical rotations.

## Methods

### Curriculum design

**Figure 1.**
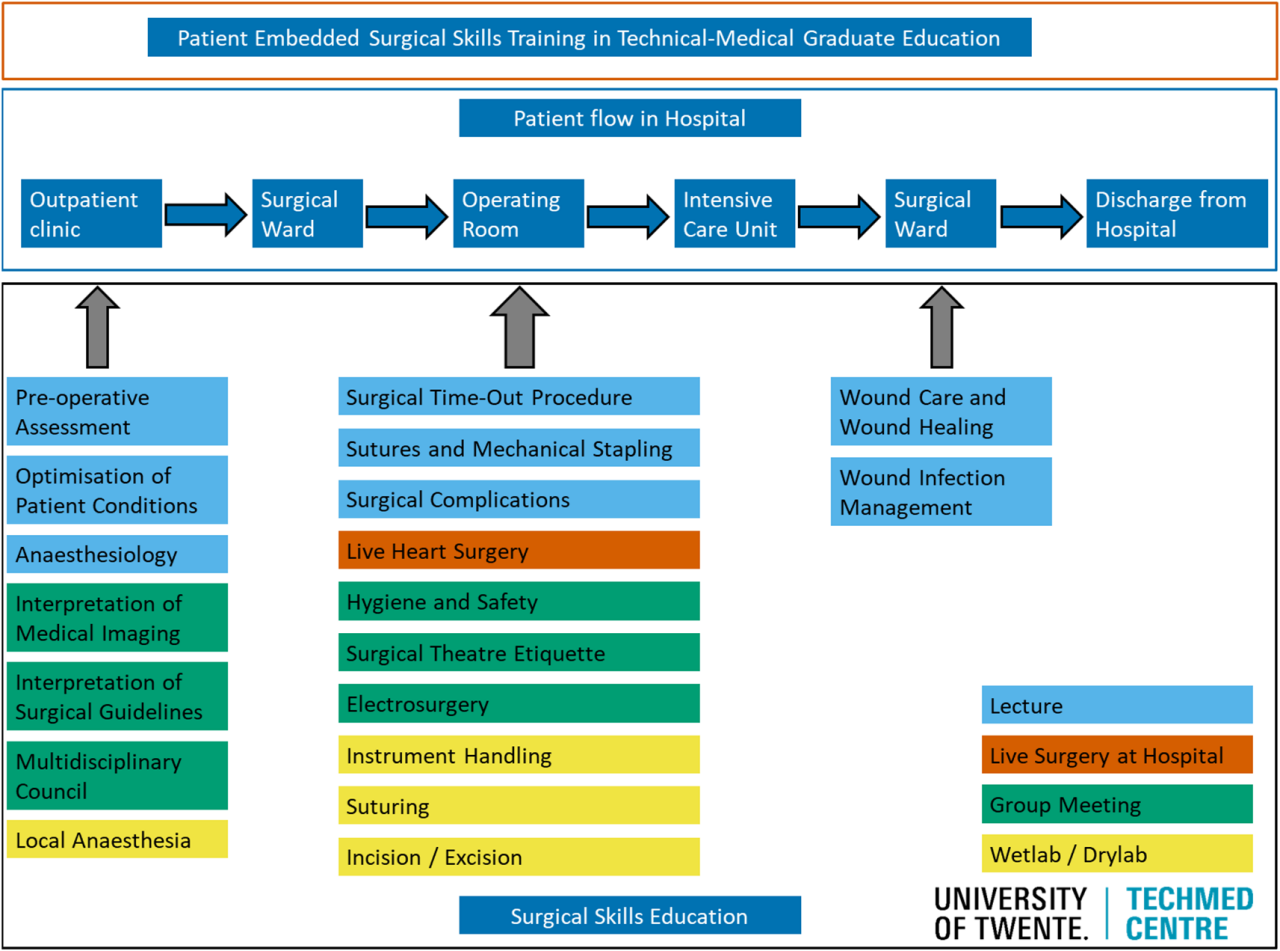
New surgical skills curriculum with patient surgical flow as backbone

A needs assessment was performed to identify learning goals ( Knowles, 1980). The main learning goal was to develop knowledge, understanding and (non) technical skills in surgery that matches daily practice. Hereafter, activities based on general patient surgical flow were developed and discussed with 4 professors in surgery and resulted in a new Surgical Skills course ( Figure 1).

The 10-week 3 European credit transfer and accumulation system (ECTS; 28 hours per ECTS credit) graduate course for Technical Medicine students is described in more detail in Supplementary File 1. An international comparison of medical education is described elsewhere ( Weggemans *et al.*, 2017). All students had prior training in non-technical communication and professional behaviour skills with simulated patients and followed a mandatory injections and catheterization skills course prior to enrolment in the surgical skills course ( Miedema, 2015; Groenier, Pieters and Miedema, 2017).

### Assessor training and evaluation

Prior to the procedural assessment, assessors were trained and interrater reliability was established for three out of four assessors as one assessor did not complete the evaluation. Each assessor evaluated four video recordings of the suturing station of a previous cohort using the rating scale developed for the current course. Two-way mixed effects single measures absolute agreement intraclass correlation coefficients (ICC), Cohen’s kappa, and proportion agreement were calculated to examine inter-rater reliability. Overall ICC for interrater reliability of the rating scales scores was low, *r* = 0.27 (95% CI = 0.03-0.54). Interrater reliability for the pass/fail scores was higher, with a mean proportion agreement of 0.75 (SE = 0.05). However, Cohen’s kappa estimates showed clear disagreements in scores when examining each pair of assessors separately. Hereafter, the rating scales were discussed with all assessors until agreement on interpretation was reached.

### Technical surgical skills assessment

A four-station procedural assessment was developed of basic surgical tasks that included scrubbing and donning, local anaesthesia, incision/excision, and suturing. In accordance with best practices ( Kottner *et al.*, 2011; Groenier *et al.*, 2020), performance indicators and objectives for the four tasks were determined by an expert panel consisting of four professors in surgery and two technical physicians in surgery. These indicators were based on national surgical residency programs and experts opinion and judged to reflect acceptable practice in the Technical Medicine training program (See Supplementary File 1).

A rubric was developed for scrubbing and donning and procedure-specific rating scales were developed for local anaesthesia, incision/excision, and suturing (example for suturing in Supplementary File 2). Critical errors, i.e. compromising patient or personal safety such as not returning sharp instruments to the instrument table or neglecting feedback of the assessor, were defined for each rating scale. Students who made a critical error immediately failed the assessment. For each station benchmarks were set for passing and students needed to pass each station to pass the entire procedural assessment.

**Figure 2.**
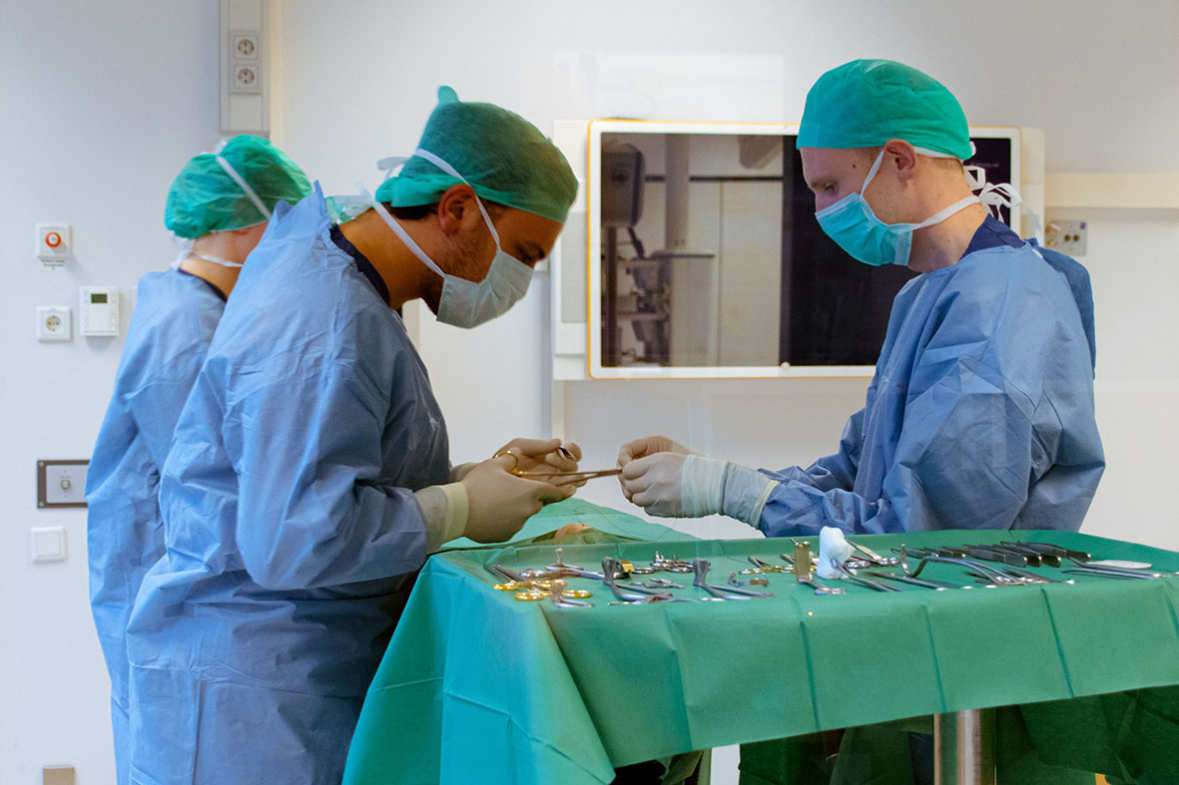
Setup of the suturing station in a simulated operating room with two examinees (left) and one examiner (right)

Two students were assessed in parallel by one assessor in blocks of 8 students spread over four stations in a 1-hour timeframe. Assessors were allowed to act as first assistant to the student without giving corrections unless deemed necessary. More than one assessor could be assigned to the same station on consecutive assessment days. Setup of the suturing station including OR etiquette and hygiene is depicted in Figure 2.

Permission was obtained for use of this photograph for publication.

### Theoretical assessment

A knowledge test was administered online (Blackboard versions October 2014 and Q4 2015, Washington, D.C., United States of America) and contained essay, closed-ended, and multiple choice questions with input from all assessors and guest lecturers. Item-difficulty (percentage of students who answered a test question correctly) and item-discrimination (the degree to which students with high overall exam scores also answered a particular question correctly) were calculated to examine reliability of the assessment.

### Post-course evaluation

A cohort study was conducted of students who received this mandatory surgical skills training in academic years 2015-2016 and 2016-2017 and was evaluated after at least one clinical rotation, depending on the start of their clinical rotations program. Students were approached by e-mail 8 months after the course. A reminder was sent three months later. The principles outlined in the Declaration of Helsinki were followed. Written informed consent was obtained from all students before the study. This research was not submitted for Ethics Board approval because this research was not subject to the Dutch Medical Research Involving Human Subjects Act (WMO). Post-hoc ethical review from the University of Twente stated having no ethical concerns regarding this research.

A one-group post-test-only design was used for post-course assessment. The Kirkpatrick model for training evaluation was applied ( Bates, 2004). Knowledge and skills acquired during the course (Level 2) were evaluated with a knowledge test and a procedural assessment. Students’ reactions to the course (Level 1) and self-reported clinical behaviour (Level 3) were measured with an online survey after finishing the course.

The online survey (SurveyMonkey, San Mateo, California, USA) consisted of statements related to confidence in own capabilities, patient safety and application of knowledge and self-reported technical skills in practice (see Supplementary File 3 for full questionnaire). Students reported the number of technical skills they performed and general questions about the course on two 6-point Likert scales.

### Statistical analysis

First, internal consistency of the knowledge test, checklist and rating scale items was evaluated. Second, an item analysis of the knowledge test items was performed. Finally, scores, grades and pass/fail rates for all assessment instruments were described and subgroup comparisons made where appropriate.

Quality of assessment instruments was evaluated using internal consistency for the knowledge test, checklist, and rating scale scores using Cronbach’s alpha and hierarchical omega ( *ω _h_*) ( Revelle and Zinbarg, 2008; Sijtsma, 2008). Both Cronbach’s alpha and hierarchical omega were used to compare estimates and possibly reduce bias and misinterpretation.

Scores, grades, and pass/fail rates for the knowledge test and skills assessment were calculated. Continuous variables were presented as mean with standard deviation (SD) or median with interquartile range (IQR) depending on the distribution. Continuous variables were tested for normality with visual inspection of histograms and skewness and kurtosis measures.

An univariate general linear model was performed to determine coherence between knowledge test and technical skills assessment. A mixed model was used to identify a potential drift in grading during assessments. Here blocks of students were created based on the 1-hour assessment blocks with 8 students.

A gender subgroup analysis as encouraged by the Institute of Medicine ( Institute of Medicine (US) Board on Population Health and Public Health Practice, 2012) was performed. Items of the online survey are presented as number with corresponding percentages and compared between cohorts and male and female students using a Fischer Exact test because less than 5 events occurred per item. A sensitivity analysis with Mann-Whitney U testing was performed by excluding the “not applicable” answer from the 6-point categorical Likert scale resulting in a 5-point ordinal scale.

## Results/Analysis

### Curriculum

The surgical skills course was redesigned based on surgical patient flow ( Figure 1). A total of 58/71 (82%) students signed informed consent for this study in 2015-2016 and 62/84 (74%) students in 2016-2017. The pass rate for the students who signed consent was similar to the full cohort of students. Table 1 shows a full overview of student population and pass rates.

**Table 1.** Student population and pass rates of the surgical skills course

	Cohort 2015-2016	Cohort 2016-2017
Students, informed consent for study	58/71 (82%)	62/84 (74%)
Female, %	39/58 (67%)	39/62 (63%)
**Pass knowledge test**	58/58 (100%)	56/59 (95%)
First examination	51/57 (89%)	40/59 (68%)
Second examination	7/7 (100%)	16/17 (94%)
**Pass first procedural assessment ^1^**	48/56 (86%) ^2^	52/59 (88%) ^3^
Scrubbing and donning	51/52 (98%)	58/58 (100%)
Local anaesthesia	53/54 (98%)	58/58 (100%)
Incision/Excision	50/52 (96%)	54/54 (100%)
Suturing skills	52/56 (91%)	52/59 (88%)

^1^ Students failing a station of the procedural assessment were not allowed to complete their assessment

^2^ One student stopped during the course, one student did not attend the assessment.

^3^ One student did not attend the assessment

A detailed overview of each station and student flow through the study are shown in Supplementary File 4 and Supplementary File 5.

### Knowledge and skill acquisition

Internal consistency for the knowledge tests, i.e. whether items of the knowledge test measured the same general construct, was poor to moderate, see Table 2, and higher for cohort 2016-2017 compared to cohort 2015-2016. Item-total correlations ranged from -0.058 to 0.445 for cohort 2015-2016 and from 0.18 to 0.55 for cohort 2016-2017, indicating that some items had poor ability to discriminate poor from good performing students, while other items had very good ability to discriminate. Internal consistency measured with omega was lower for both tests compared with Cronbach’s alpha and less sensitive to removing poor performing items.

Internal consistency was acceptable to high for suturing for both Cronbach’s alpha and omega. It was fair to good for incision/excision and poor to acceptable for local anaesthesia and this varied depending on the internal consistency measure used, see Table 2. For scrubbing, Cronbach’s alpha was very small and negative for the long version including all 11 items, indicating negative inter-correlations between the individual items. Univariate analysis showed no significant prediction of technical skills score from the knowledge test, F(1,113)=3.335, p=0.07.

In 2015-2016, significant lower grades were given on the second assessment day (Thursday) for scrubbing and donning (10.0 vs 9.0, U=182, p=0.005), local anaesthesia (7.0 vs 8.0, U=181, p=0.001) and excision/incision (8.0 vs 7.5, U=220.5, p=0.04). A year later, both local anaesthesia (7.0 vs 7.5, U=289, p=0.03) and excision/incision (7.0 vs 7.5, U=210.5, p=0.009) scored lower on the second assessment day in 2016-2017.

**Table 2.** Quality of assessments of procedural assessment and knowledge test

	Cohort 2015-2016		Cohort 2016-2017	
	Cronbach’s alpha	Omega ( *ω _h_*)	Cronbach’s alpha	Omega ( *ω _h_*)
**Knowledge test**				
Original test (21 questions in 2015-2016, 18 questions in 2016-2017)	0.48	0.36	0.78	0.36
Test 2015-2016, without poor performing items (8 questions)	0.66	0.31	-	-
**Procedural assessment**				
Scrubbing and donning	-0.01	N/A ^1^	-0.29	N/A ^1^
Local anaesthesia	0.55	0.56	0.71	0.43
Incision/Excision	0.85	0.67	0.63	0.41
Suturing skills	0.94	0.85	0.79	0.54

^1^ Four items had no variation and hierarchical omega ( *ω _h_*) could not be calculated for all 11 items of the scrubbing checklist. These items were removed from further analysis.

An overview of median grades per skill assessment station is given in Table 3. There was no difference in grading between timeslots within a day (no rater drift) as determined with mixed model analysis (Supplementary File 6). Only the 2016-2017 excision/incision assessment on Thursday showed a significant drift (Supplementary File 6, Figure D, F(4,23)=4.388, p=0.009).

**Table 3.** Median grades per skills station per assessment day for both cohorts

	2015-2016			2016-2017		
	Day 1	Day 2	P-value	Day 1	Day 2	P-value
**Scrubbing/donning**	10 [9-10]	9 [8-10]	0.005	10 [9-10] ^1^	10 [9-10] ^1^	0.57
**Local Anaesthesia**	7 [6.5-7.5]	8 [7.5-8]	0.001	8 [8-8]	7.5 [7-8]	0.03
**Excision/Incision**	8 [7-9]	7.5 [6-8]	0.04	7.5 [7-8]	7 [6.75-7.5]	0.009
**Suturing**	7.75 [7.38-8.5]	8 [7-8]	0.54	8 [7-8]	8 [7-8]	0.70

Median [IQR]

^1^ Scrubbing and donning was transformed from a grade to a pass/fail criterium in 2016-2017 where 100% of the students obtained a pass.

### Results post-course questionnaire

Out of 71 students from cohort 2015-2016, 27 (38%) filled in the post-test questionnaire after 1 [1-2] clinical rotation. For cohort 2016-2017, 21/84 (25%) filled in the questionnaire after 3 [2-3] rotations. Mean age was 23 ± 1.1 years and 69% was female and is in line with the male to female ratio of medical students in the Netherlands ( Erasmus *et al.*, 2020).

**Figure 3.**
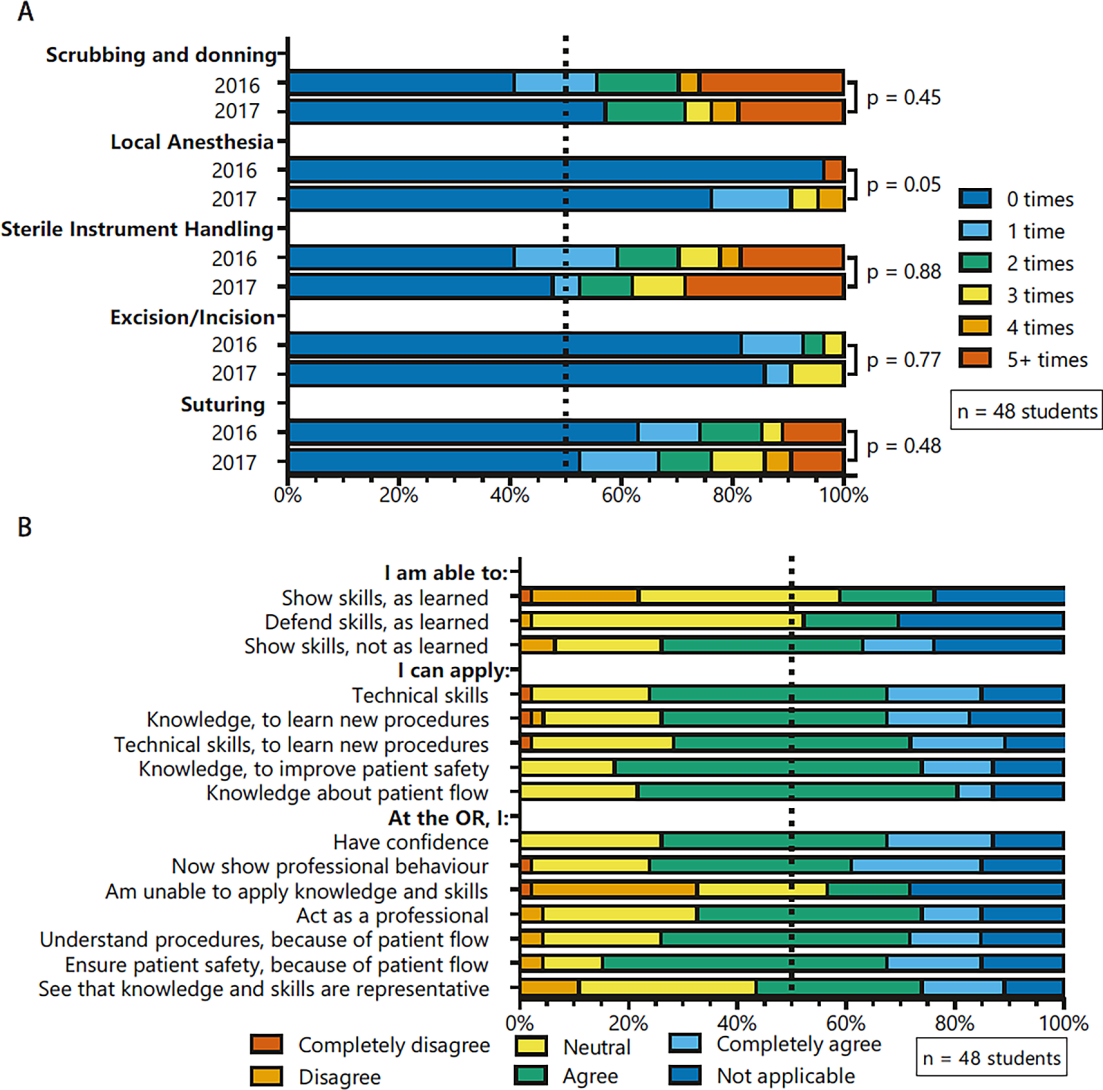
Post-course questionnaire 2015-2016 (n=27) and 2016-2017 (n=21)

Most students handled sterile instruments during their clinical rotations (52-59%), whereas performing local anaesthesia (4-24%) and incision/excision (14-18%) were rare, see Figure 3A. There was no significant difference in number of procedures between cohorts, except for local anaesthesia (U=229,p=0.05) where more procedures were performed in cohort 2016-2017.

Female students performed a higher number of technical skills than male students for scrubbing and donning (U=160, p=0.04), sterile instrument handling (U=160.5, p=0.04), incision/excision (U=187.5, p=0.04) and suturing (U=168.5, p=0.049) but not for local anaesthesia (U=202.5, p=0.08), see Supplementary File 7.

**A** Amount of technical surgical skills performed in early clinical rotations **B** Course feedback of students during clinical rotations. OR=operating theatre

In ordinal regression analysis, the odds of male students reporting fewer frequencies for scrubbing and donning were 1.40 (95% CI, 0.12 to 2.7) times that of female students, a statistically significant effect, Wald (χ2 (1) = 4.595. The odds for reporting fewer frequencies by male students for suturing were 1.41 (0.001 to 2.8), p=0.05, and 1.30 (0.08 to 2.5), p=0.04 for sterile instrument handling. Male students reported not performing incision/excision or local anaesthesia during their clinical rotations.

### Preparation for clinical practice

Only 10% of the students received OR introduction at all clinical rotations, while 10% received this sometimes and 58% never received an instruction. Others described that they not yet attended the OR (17%) or received more tailored instructions (4%).

There was no significant difference in course feedback questions between cohorts. Most students were confident about attending a surgery and agreed about having a professional attitude in the operating room, see Figure 3B. Furthermore, students asserted that knowledge on patient safety is important. Half of the students were able to acquire new variations on technical skills.

In the sensitivity analysis using ordinal coding, a significant higher proportion of cohort 2015-2016 reported that they were unable to apply knowledge and skills at the OR, compared to cohort 2016-2017 (U=84, p=0.03). There was no significant difference in course feedback questions between cohorts for the other questions.

Two thirds of the male students reported they are able to act as a professional (0% completely agreed, 67% agreed), while female students responded more neutral (16% completely agreed, 29% agreed), p=0.02 (Cramer’s V=0.47). The sensitivity analysis confirmed this finding (U=105.5, p=0.03) and demonstrated that male students attend the OR with more confidence than female students (U=117, p=0.03). No other statistically significant differences in responses between male and female students were found.

## Discussion

Proficiency-based, simulation-based surgical skills training is recommended, however, implementation is not always straightforward ( Hosny *et al.*, 2017). In this paper, a balanced basic surgical skills curriculum was described that adequately prepared students for their clinical rotations.

The surgical skills curriculum was anchored to surgical patient flow focusing on knowledge, technical and non-technical surgical skills. Also, assessors were trained to prevent biased judgments before they assessed students on basic surgical skills. Afterwards, sterile instrument handling, scrubbing and donning, and suturing skills were often performed during clinical rotations. The majority of students felt confident during their clinical rotations about attending a surgery and stated they could show a professional attitude in the operating room.

### Curriculum redesign

The main learning goal of this course was to develop knowledge, understanding and (non) technical skills in surgery that matches daily practice. Graduate students were able to reach proficiency level in a simulation-based basic surgical skills course. Students stated to go with confidence to the operating room and felt competent in performing four basic surgical skills.

Most residency programs assess surgical skills at the end of the rotations and generally with subjective evaluations ( Bhatti, 2017), while these skills were already performed on patients. Furthermore, surgical skills are not acquired by all students and many medical students are not able to show sufficient surgical skills after completing a 3 ^rd^-year surgery clerkship ( Pender *et al.*, 2017). This surgical skills course is compulsory next to other skills courses on vein-puncture and peripheral catheterization, advanced life support and endoscopic skills. This curriculum might be more difficult to apply for programs without these skills courses. Nevertheless, aspects of this curriculum following surgical patient flow are usable for all programs. A minimum quality of surgical skills in a mandatory simulated environment is guaranteed for all students with a pass on the surgical skills course described in this study.

The redesign of our SBT was in accordance with three out of four principles described elsewhere ( Zevin, Aggarwal and Grantcharov, 2014), to be mandatory participation, proficiency-based, and a distributed training schedule. Our SBT lacked a component of overtraining. This seems acceptable at graduate level for Technical Medicine students, because the program aims for adaptive rather than routine skills development ( Groenier, Pieters and Miedema, 2017). In retrospect, the principles of developing and refining a surgical curriculum as recently described were followed ( Sell and Phitayakorn, 2020).

Based on internal course evaluations, local anaesthesia wet lab time was reduced (academic year 2017-2018) because Technical Medicine students tend not to perform these skills during rotations. More recently, more time was allocated to post-operative care such as wound healing (2018-2019) and enhanced recovery after surgery (2019-2020).

### Course redesign evaluation

Surgical knowledge as deemed necessary by assessors was tested in a theoretical assessment. The majority of students passed this test on first examination (68-89%) and almost all students on second examination (94-100%). For the two cohorts described in this paper, technical skills were outstanding ( Netherlands Organization for International Cooperation in Higher Education (NUFFIC), 2009) for scrubbing and donning (10 [9-10]), good for local anaesthesia (8 [7-8]), very satisfactory to good for incision/excision (7.5 [7-8]) and good for suturing (8 [7-8]).

Internal consistency was poor to acceptable for local anaesthesia, fair to good for incision/excision and acceptable to high for suturing. For scrubbing, Cronbach’s alpha was very small and negative because of little variation for the majority of items (see Table 2).

A possible bias in pass/fail decisions for the course was compensated by testing knowledge and skills separately with equal weighing, by using multiple assessors, and by assessing a variety of surgical skills (van der Vleuten and Schuwirth, 2005). To improve reliability, we propose more rigorous and repeated assessor training and exclude assessors who do not meet the standards for examination.

In this study around 80 students per academic year were assessed for technical skills in 1-hour blocks of maximum 8 students for two days by four unique assessors. This study is among the largest study groups described ( Bevilacqua *et al.*, 2020). A total of 48 man-hours was necessary for assessment because of parallel assessment of students. The 3 ECTS course was time consuming only to two course coordinators and to a lesser extent for guest lecturers. This curriculum is thus suitable for large student cohorts.

Previous work suggested rater drift between assessment sessions ( Gawad *et al.*, 2019). No drift in grading was observed for all skills stations in both cohorts except for excision in 2016-2017 on day 2. The impact of assessor training is reduced as early as 4 months after in-depth training ( Gawad *et al.*, 2019) and may have resulted in more drift during the second academic cohort. Annual training before starting the course is thus recommended for consistent assessment.

### Post-course evaluation and student perceptions

An import aspect of SBT is to give confidence to learners. The majority of students in this study stated to go with confidence to the operating room and felt competent in performing four basic surgical skills. Some studies have used validated confidence scores ( Bevilacqua *et al.*, 2020), while a wide range of questions were asked in this study ( Figure 3B). Validated confidence scores should be implemented pre- and post a surgical skills course.

Students struggled most with clinical reasoning for complex cases and taking patient safety into account while performing a basic procedure. Furthermore, students reported that they were least confident about performing basic surgical tasks during their clinical rotations the way they were taught ( Figure 3B). Demonstrating more surgical technique variations in daily practice might improve adaptivity of students.

### Limitations and possible improvements

Many surgical education studies show confounders and lack a pre-test, comparator or relevance ( Corsini and Antonoff, 2020). Students in Technical Medicine had no expected previous surgical skills experience and a pre-test on technical skills was not deemed necessary. A validated pre- and post-test on self-reported confidence ( Bevilacqua *et al.*, 2020) could be implemented to improve course evaluation. This study did not compare to the previous surgical skills curriculum or other programs. Therefore, effects on knowledge and skills acquisition cannot with certainty been attributed to our redesign only. Finally, it is likely that passing this course results in safe patient care as students demonstrated all-round technical and non-technical surgical skills making this course highly relevant.

The effects of redesign on patient outcomes (Kirkpatrick Level 4) were not evaluated. The self-reported clinical performance (Kirkpatrick Level 3) has limitations. Future studies should ask surgeons in a pre- and post-questionnaire on differences in clinical behaviour and skill transfer of students. Patient-centred medical education should be about patients, with patients and for patients ( Hearn *et al.*, 2019). Simulated patients are embedded in undergraduate Technical Medicine education. This course is about and for patients and has a patient surgical journey as backbone, but needs patient participation such as simulated patients for surgical ward training.

Gender subgroup analysis is endorsed by many organizations and studies ( Institute of Medicine (US) Board on Population Health and Public Health Practice, 2012; Bevilacqua *et al.*, 2020). An unplanned gender analysis showed more procedures in female students for all technical skills except for local anaesthesia which was seldom performed among all students (Supplementary File 7). An interaction was found between gender and technical skills in ordinal regression analysis with fewer reported frequencies in scrubbing and donning, sterile instrument handling and suturing. No difference was found in the behavioural questions except for acting as a professional where female students answered more neutral. These gender differences are in contrast with others where lower scores in self-efficacy for female students disappeared after a surgical boot camp ( Bevilacqua *et al.*, 2020). Future cohort studies should include prespecified and well-designed ( Barraclough and Govindan, 2010) gender subgroup analysis to investigate potential gender related differences.

## Conclusion

Acquiring basic surgical knowledge and skills is feasible in a 10 week, 3 ECTS, simulation-based and proficiency-based course for graduate students. Trained and committed faculty, mandatory participation, and a proficiency-based program, determine the effectiveness of a course to a large extent.

By evaluating the course at the level of knowledge, skills and students’ perceptions, areas for improvement of a basic surgical skills course at the graduate level were identified and applied to following courses.

## Take Home Messages

• A new graduate surgical skills curriculum was anchored to surgical patient flow
• The course focuses on knowledge, technical and non-technical surgical skills
• Assessors were trained to prevent biased judgments on basic surgical skills
• Students felt confident during their clinical rotations about attending a surgery
• Students should be proficient in surgical skills before practice on patients

## Notes On Contributors

Dr. Frank R. Halfwerk is a Technical Physician in cardio-thoracic surgery at Medisch Spectrum Twente and Assistant Professor at TechMed Centre, University of Twente. ORCiD: https://orcid.org/0000-0003-2928-9728

Dr. Erik Groot Jebbink is a Technical Physician in vascular surgery at Rijnstate Hospital and Assistant Professor at TechMed Centre, University of Twente. ORCiD: https://orcid.org/0000-0001-7041-8603

Dr. Marleen Groenier is a human factors psychologist and coordinator of technical-medical educational research at TechMed Centre, University of Twente. ORCiD: https://orcid.org/0000-0001-7298-8431
