# Supplementary material for: Development and Evaluation of a Proficiency-based and Simulation-based Surgical Skills Training for Technical Medicine Students
Source: MedEdPublish (2016). 2020 Dec 17;9:284. [Version 1] doi: 10.15694/mep.2020.000284.1 (PMC10697565; doi:10.15694/mep.2020.000284.1)
Supplement: Peer review history

# Reviewer report: Reviewer response for version 1

**DOI:** 10.21956/mep.20185.r31218

**Published:** 2021-07-13

**Authors:** Mildred López

**Affiliations:** 1 Tecnologico de Monterrey, School of Medicine and Health Sciences

This review has been migrated. The reviewer awarded 4 stars out of 5 I think the implementation of the technical medicine curriculum is an experience that more educators would find valuable. As the other reviewer mentioned not everyone is familiar with the program, the authors could help readers from other contexts to understand the program by including a description in the design of the curriculum or the introduction section. I would recommend separating parts A and B from figure 3 and have two figures.

Reviewer Expertise:

NA

No decision status is available

# Reviewer report: Reviewer response for version 1

**DOI:** 10.21956/mep.20185.r31219

**Published:** 2021-05-25

**Authors:** Willem Brinkman

This review has been migrated. The reviewer awarded 3 stars out of 5 This manuscript describes the work of the authors of the development of a scientifically based proficiency based surgical skills curriculum. Congratulations to the authors on the manuscript. The curriculum has a solid ground with a needs-analysis with experts in the field. The data is well presented and clear and the tables and figures are supportive. As already indicated by the authors, it is very unfortunate that no comparison is possible with the data obtained before these adjustments. Also a comparison with a program described by others is not performed and that seriously limits the scientific value of this manuscripts. I understand that these data are not available and therefore this cannot be improved. Another aspect that can be improved is to add some information about the general technical medicine curriculum and the learning objectives of the surgical rotation. Not everyone is familiar with the technical medicine curriculum and the expected outcome of their rotation. Is there literature by others about the technical medicine curriculum? If not this can be highlighted in the manuscript that this is the first description.

Reviewer Expertise:

NA

No decision status is available

# Reviewer report: Reviewer response for version 1

**DOI:** 10.21956/mep.20185.r31220

**Published:** 2020-12-21

**Authors:** Leila Niemi-Murola

**Affiliations:** 1 University of Helsinki

This review has been migrated. The reviewer awarded 3 stars out of 5 Thank you for the opportunity to review the interesting manuscript focusing on improvement of basic surgical skill using a simulation-based course. The authors included a wide scope of skills in their course, not just hands-on skills. In the introduction, the authors have motivated the need for this course. However, the educational theoretical basis of the course remains vague.In the Methods section, the authors present the outline of the course, combining successfully theory and practice in the OR. The assessors were trained in order to develop a shared mental model of the participants’ performances. The authors have included the rubrics used as tools of assessment as the supplements. The one used for assessment of local anaesthesia and suturing resembles the OSATS. Theoretical assessment was made by used by using an online-test, but this has not been included as a supplement. Thirty-eight percent of the participants filled in the first questionnaire, and 25 % filled in the questionnaire after the three rounds. This is not an unusual response rate for electronic questionnaires.The course was based on needs assessment, but the authors have not described this pilot phase of the study. In the results, the authors give the results of two cohorts, years 2016 and 2017. The results of the year 2017 were statistically significantly better than those of the 2016 students. However, it was not quite clear to me if the students in the former study year had received the same education as the students of the latter and if they were, was the course modified after the first round.The paper describes a very inspiring educational intervention and the results are encouraging.

Reviewer Expertise:

NA

No decision status is available
